# Supplementary material for: Perceived causes of marital dissatisfaction among Nigerian immigrants in North America: A qualitative study
Source: PLoS One. 2025 Aug 7;20(8):e0327551. doi: 10.1371/journal.pone.0327551 (PMC12331035; doi:10.1371/journal.pone.0327551)
Supplement: Attachment — Submitted filename: Response to reviewers.docx [file pone.0327551.s002.docx]

**Response to Reviewers**

Reviewer 1:

We thank you for taking the time to review our manuscript and for your thoughtful feedback. Our responses to your feedback are numbered according to the numbering in your feedback and as follows:

**Thanks for your kind review.**

Issues for consideration in the revision and for further studies:

1. **This research recruited participants from two countries with dissimilar socio-cultural and immigration practices. Could these contextual factors bias the results? This study could be further strengthened by highlighting the nuances in the two socio-cultural milieus in which the participants live.**

Thank you for this insightful observation. While the study involved Nigerian immigrants residing in two different North American countries, we note that both settings share comparable socio-cultural frameworks, particularly regarding family structure, individual autonomy, and immigrant integration policies typical of Western societies (Nevitte et al., 2017). This commonality is reflected in the participants’ experiences of life and their conceptualization of marital dissatisfaction.

A review of the literature did not reveal substantial differences in socio-cultural or immigration practices between the two countries that would significantly influence perceptions of marital dynamics among Nigerian immigrants. Furthermore, during the data analysis phase, responses from participants across both countries were thematically consistent, with no notable divergence in the perceived causes of marital dissatisfaction. This thematic uniformity suggests that the shared immigrant identity and cultural background of the participants may have had a stronger influence on their marital experiences than their country of residence.

Nevertheless, we recognize the importance of contextual nuance and have revised the manuscript to briefly acknowledge the potential for socio-cultural variation, while justifying our decision to present the findings collectively based on the observed homogeneity in responses (pages 7 & 39). We believe this approach maintains the integrity of the study’s qualitative focus while addressing the reviewer’s valid concern.

2. **The socio-demographic variables show a heterogeneous population. It is important to note this as a potential source of bias in the reporting.**

Thank you for this observation. We acknowledge that the participants represented a heterogeneous group in terms of age, length of residence in North America, marital duration, and educational backgrounds. However, this diversity was intentionally included to capture a broad spectrum of lived experiences among Nigerian immigrants. In qualitative research, such heterogeneity is not typically viewed as a source of bias, but rather as a strength that enhances the richness, depth, and transferability of the findings.

We used purposive sampling to ensure variation in socio-demographic characteristics, allowing us to explore how marital dissatisfaction may be perceived differently across various subgroups within the immigrant population. Importantly, the thematic analysis revealed consistent patterns across the diverse sample, suggesting shared cultural and contextual influences that transcend individual differences.

*Nonetheless, we have revised the manuscript to acknowledge this heterogeneity as a potential limitation in terms of the specificity of subgroup comparisons (page 40).*

3. **More so, some participants, in addition to their different socio-cultural and economic factors, presented with different marital problems. It is expected that participants with troubled relationships would normally not open up in a counseling session, let alone during an interview. Although not clear from the researcher’s profile information, there seems to be a minor gender difference between the participants and the researchers. It is interesting how the researchers handled the sensitive nature of the study. Did the researchers interview participants of the same gender? What were the gender considerations? How did the researchers ensure that the information was given in a relaxed atmosphere? These types of participants should be interviewed by trained personnel and, thereafter, for those in need of help, offered some social support after the research. How these were addressed by the authors is important.**

Thank you for raising this important point. We acknowledge that marital dissatisfaction is a sensitive topic and took several steps to ensure participants were emotionally safe and comfortable during data collection. All interviews were conducted by trained female interviewers who were culturally competent and had experience working with immigrant communities. One of the interviewers is a mental health provider. Despite the potential for gender-based discomfort, participants reported feeling at ease during the interviews and were able to express themselves openly. Many expressed appreciation for the opportunity to share their experiences in a supportive and non-judgmental setting.

Interviews were conducted via Zoom, allowing participants to remain in the privacy of their own homes, which helped minimize distractions and foster a relaxed environment. To enhance anonymity and reduce self-consciousness, video features were not used during the interviews. This approach allowed participants to speak freely without concern about visual identification.

Additionally, as part of our trauma-informed research protocol, participants were informed ahead of time that they could pause or stop the interview at any point. At the conclusion of each interview, they were provided with a list of culturally relevant counseling and support resources in case the conversation triggered emotional distress or re-traumatization. While our study was not a therapeutic intervention, we ensured that participants were informed about the researchers’ availability to provide mental health referrals and counseling as needed.

*We have revised the Data Collection section of the manuscript to reflect these important methodological safeguards (page 8).*

4. **The age at which couples experience marital instability may not be in their early years, at least from empirical and anecdotal reports. So, it is surprising to see that the participants were recruited from a seemingly young (first year) marital age. Could the findings have shown a different view if those with more years of marriage were interviewed?**

Thank you for this thoughtful observation. We agree that marital dissatisfaction can emerge at various stages of a marital relationship and that couples who have been married for longer durations may experience different or more complex challenges. However, our study intentionally included participants at various stages of marriage, including those within the early years. In the immigrant context, early marital instability is not uncommon due to the compounding effects of acculturative stress, shifting gender roles, financial strain, and limited social support—factors that may intensify in the initial years post-immigration.

Our inclusion of participants within the early years of marriage aimed to capture these formative experiences and the ways in which migration-related factors impact marital expectations and satisfaction from the onset. While it is possible that couples married longer may have developed different coping mechanisms or perspectives, our thematic analysis revealed patterns of dissatisfaction that were consistent across different marital durations. Moreover, the mean duration of our participants’ years of marriage is 19.71 years (see Table 2).

5. **Using audio instead of a video zoom interview may worsen the inherent challenges of maintaining engagement, observing non-verbal cues, and other technological issues. How these were considered in the study design should be highlighted.**

Thank you for raising this important concern. We agree that video interviews offer advantages in capturing non-verbal cues and fostering rapport. However, for this study, we made a deliberate choice to conduct audio-only Zoom interviews in order to protect participant anonymity and increase their willingness to share openly, especially given the sensitive nature of the topic—marital dissatisfaction. This approach is supported by existing literature, which suggests that audio-only interviews can facilitate greater openness and reduce self-consciousness when discussing personal or stigmatized experiences (Oates et al., 2022).

To address potential challenges with engagement and data quality, our interviewers were trained in verbal rapport-building techniques and active listening strategies to maintain participant engagement throughout the session. These included reflective prompts, verbal affirmations, and periodic summarizing to confirm understanding and encourage deeper elaboration. We also scheduled interviews at times chosen by participants to minimize distractions and ensure focus. While non-verbal cues were limited, the depth and clarity of participant responses remained strong, and saturation was achieved during thematic analysis.

6. **The paper should report how privacy (data privacy) and confidentiality issues were handled during and after the Zoom audio interview. Third-party Zoom handling of data and how the researchers and participants maintain privacy and confidentiality while using an audio interview should be reported.**

Thank you for this important point. We took several precautions to ensure participant privacy and data confidentiality throughout the study, especially given the use of a third-party platform like Zoom. First, all Zoom interviews were conducted using institutional Zoom accounts licensed through our university, which are protected by end-to-end encryption and adhere to U.S. FERPA and HIPAA standards. These accounts do not allow data to be stored on Zoom’s cloud servers; instead, all recordings are saved directly to secure, encrypted university servers.

Second, interviews were conducted without video and without the use of participant names or identifying information during the session. Participants were assigned pseudonyms used in all transcripts and analysis files. Interviews were scheduled at private times and locations determined by participants to further ensure confidentiality.

All data files, including audio recordings and transcripts, were stored in encrypted folders on password-protected devices accessible only to the research team. No data were shared with third parties. Additionally, all members of the research team completed human subjects training and signed confidentiality agreements approved by the Institutional Review Board (IRB).

*We have revised the Ethical Considerations section of the manuscript to reflect these privacy safeguards (page 12).*

Reviewer #2:

**Main Observations**

7. Clarify marital dissatisfaction

**I will suggest that you clearly define what constitutes marital dissatisfaction (e.g., routine stress versus abuse or emotional disconnection). For example, the information provided in line 536 suggests exhaustion of the participant rather than dissatisfaction in their marriage. Ensure consistency between the conceptual framework and participant responses to strengthen the study’s focus.**

Thank you for this insightful recommendation. We agree that providing a clear and consistent definition of marital dissatisfaction is essential to strengthening the conceptual clarity of the study. In this study, we adopted a broad, participant-driven understanding of marital dissatisfaction as referring to any ongoing negative perception or experience within the marital relationship that undermines emotional and physical well-being, fulfillment, or a sense of connection. It may involve emotional disconnection, unmet expectations, gender-role conflicts, poor communication, or cumulative stress resulting from interpersonal dynamics or structural factors (Mota & Sanchez, 2023; Randall & Bodenmann, 2017).

Regarding the example noted in line 536, we appreciate the observation and have clarified in the revised manuscript that the participant’s reported exhaustion was described in the context of their perception of recurring marital responsibilities and lack of partner support, both of which contributed to a sense of imbalance and emotional burden in the relationship. We recognize that while physical fatigue alone may not constitute dissatisfaction, in this case, it was part of a broader pattern of frustration and perceived inequity within the marriage.

*To address this concern, we have revised the manuscript to include the participant-driven operational definition (page 4).*

8. Expand participant details

**The methodology adopted for the study is robust and thoughtfully considered. However, it would be beneficial to include participants’ occupations and their personal experiences with marital dissatisfaction. Clarify whether participants are direct experiencers or observers (e.g., counselors, clergy), as this impacts the credibility of their insights. This context would support the quotes used to illustrate the identified themes. The omission of these factors in the inclusion criteria seems to undermine the study’s findings, especially since you frequently reference the lived experiences of participants (lines 104, 115) and their observations (lines 239, 434).**

Thank you for your thoughtful comment. We appreciate the opportunity to clarify the inclusion criteria and participant roles in this study. Participants were eligible if they had either personally experienced marital dissatisfaction or had closely observed it in others, such as family members or close friends. This approach was informed by the understanding that lived experiences within immigrant communities are often shared collectively, and observations, particularly within extended family or social networks, can deeply inform an individual’s understanding of marital dynamics.

It is important to note that none of the participants were included based on professional roles, such as counselors, clergy, or service providers. During the interviews, no participant identified themselves as speaking from a professional perspective, nor did their responses suggest professional detachment or interpretation. All accounts were given in the context of personal or relational experience, consistent with the phenomenological framework of the study.

Regarding occupational background, we did not systematically collect detailed employment data, as it was not a central variable in our study design.

9. Methodology

**The study’s qualitative sample size is relatively small to determine the prevalence of marital dissatisfaction (line 136), particularly since none of the participants reported major issues in their marriage (Table 1). Without information on the selected participants’ marital dissatisfaction or their direct interactions with couples facing marital challenges, it is difficult to ascertain the contributory factors associated with marital dissatisfaction (line 137), especially if the participants neither experience marital dissatisfaction nor hold positions of authority such as marriage counsellors, marital legal advisers, or religious leaders who may have direct and open interaction with couples facing marital challenges.**

**However, these observed limitations may not be regarded as significant because the title of the article suggests that the study is on the 'perceived' causes of marital dissatisfaction. Nevertheless, for consistency, sentences that reference lived experiences and observations should be revised to align with the title of the article.**

Thank you for this detailed and constructive feedback. We would like to clarify that the purpose of this study was not to determine the prevalence of marital dissatisfaction, but rather to explore participants’ perceptions of its causes among Nigerian immigrants in North America using a qualitative, exploratory approach. We agree that the sample size is not intended to be generalized in a statistical sense, but rather to offer rich, in-depth insights into cultural and relational perceptions of marital strain.

As noted earlier, participants were selected based on either personal experience with marital dissatisfaction or close observation of such experiences in their immediate social networks. Our intent was to explore the beliefs, understandings, and attributions individuals make regarding the factors contributing to dissatisfaction, rather than to rely exclusively on professional insight or severe marital crisis. Additionally, while some participants may not have reported "major" marital issues as defined clinically, many described patterns of emotional disconnect, role conflict, and unmet expectations, which informed their perceptions of dissatisfaction.

*We appreciate the reviewer’s observation regarding terminological consistency. To align more closely with the study’s title and objectives, we have revised sections of the manuscript to reflect a consistent use of terms such as “perceived causes,” “participant perceptions,” and “narratives of observed or experienced dissatisfaction,” and we have limited the use of “lived experience” to cases where participants clearly described their own marital dissatisfaction.*

10. Discussion: Areas for improvement

**While the study strongly critiques patriarchal structures and gender dynamics within Nigerian immigrant marriages, it does not sufficiently acknowledge cases where traditional values might contribute to marital stability, as you have noted in your earlier studies (Ade-Oshifogun, Jochebed B. et al, 2019). A more balanced discussion that incorporates instances of successful cultural integration or adaptive strategies would strengthen the discussion, especially with regard to why, despite the observed factors associated with marital dissatisfaction, Nigerian marriages are still stable with a high degree of marital satisfaction, as already established in your previous study.**

Thank you for this important observation. We agree that a more balanced discussion acknowledging the stabilizing role of traditional cultural values, as well as examples of successful adaptation within Nigerian immigrant marriages, would strengthen the interpretation of our findings.

The focus of the current study was on marital dissatisfaction, not marital stability, among Nigerian Immigrants in North America (NINA). In our previous study, we examined marital satisfaction and highlighted the limitations of existing tools in capturing the cultural nuances of marital relationships among NINAs. Participant reports in the current study did not support traditional values, such as patriarchy, as contributing positively to marital stability. Therefore, we respectfully disagree with the suggestion to include this perspective in the discussion section.

11. **Secondly, your discussion presents Nigerian men as predominantly resistant to change and women as universally oppressed or seeking empowerment. While these trends may be common, acknowledging variations (such as men who adapt egalitarian norms or women who prefer traditional roles) would provide a more nuanced discussion. Furthermore, the claim in lines 414 to 419 that Nigerian women are more likely to secure better-paying jobs than their husbands requires substantiation from relevant literature. Is there any explanation for the differentials – is it that Nigerian women have superior qualifications and more experience than their male counterparts? Indeed, various reports have suggested that the median Nigerian household income is higher than the total population in the United States (United States Census Bureau, 2023). Such information needs perceptive discussion within the overall framework of the study.**

We sincerely appreciate this valuable feedback. We agree that it is essential to avoid overgeneralization and present a balanced, nuanced interpretation of gender dynamics within Nigerian immigrant marriages.

First, regarding the portrayal of Nigerian men and women, the thematic emphasis on male resistance to change and female pursuit of empowerment was derived directly from participant narratives.

Second, regarding the claim that Nigerian women are more likely to earn more than their husbands (lines 414–419), we acknowledge the need for clarification. This statement is based on participants’ personal accounts and should not be interpreted as a generalizable or statistically verified conclusion. We have revised the language to reflect that this reflects a perceived or experienced dynamic within the sample, not an empirically confirmed trend.

We appreciate the reviewer’s reference to U.S. Census Bureau (2023) data, which shows that Nigerian households in the U.S. have a median income above the national average, indicating strong educational and professional outcomes for this population. Based on participants’ reports of Nigerian women possessing greater earning power than their male spouses, it is plausible that this higher median income may, in part, be attributed to the income contributions of women in NINA households.

12. Repetitive points and redundancy

**Some themes, particularly norms and gender role conflicts, are reiterated multiple times in similar ways. Consider these points for conciseness to improve readability and maintain reader engagement.**

Thank you for this helpful suggestion. We agree that maintaining conciseness and avoiding unnecessary repetition is essential for reader engagement and clarity. The repetition of themes about cultural norms and gender role conflicts highlights their frequent presence in participant narratives and shows how these issues appeared in different relational contexts.

However, in response to your feedback, we have carefully reviewed the Results and Discussion sections, separating the discussion from the results of each theme. We believe these edits enhance the manuscript’s readability while preserving the depth and richness of the participants’ perspectives.

**References**

Mota, C., & Sanchez,. (2023). Indicators Related to Marital Dissatisfaction. *Healthcare*, *11*(13), 1959. https://doi.org/10.3390/healthcare11131959

Nevitte, N., Nevitte, N., Basáñez, M. E., & Inglehart, R. (1996). *The North American Trajectory: Cultural, Economic, and Political Ties among the United States, Canada, and Mexico*. Taylor and Francis.

Oates, M., Crichton, K., Cranor, L., Budwig, S., Weston, E. J. L., Bernagozzi, B. M., & Pagaduan, J. (2022). Audio, video, chat, email, or survey: How much does online interview mode matter? *PLOS ONE*, *17*(2), e0263876. https://doi.org/10.1371/journal.pone.0263876

Randall, A. K., & Bodenmann, G. (2017). Stress and its associations with relationship satisfaction. *Current Opinion in Psychology*, *13*, 96–106. https://doi.org/10.1016/j.copsyc.2016.05.010
